# Phenotypic and molecular characterization of beta-lactam resistant Multidrug-resistant Enterobacterales isolated from patients attending six hospitals in Northern Nigeria

**DOI:** 10.1038/s41598-023-37621-z

**Published:** 2023-06-26

**Authors:** Nubwa Medugu, Isabella A. Tickler, Carissa Duru, Ruth Egah, Abu Ocheiku James, Vivian Odili, Fatima Hanga, Eyinade Kudirat Olateju, Binta Jibir, Bernard E. Ebruke, Grace Olanipekun, Fred C. Tenover, Stephen K. Obaro

**Affiliations:** 1 Nile University of Nigeria, Cadastral Zone, Research and Institutions Area, Abuja, Nigeria; 2 International Foundation Against Infectious Disease in Nigeria, Dutse Street, Gwarinpa, Abuja, Nigeria; 3 Cepheid, Sunnyvale, CA 94089 USA; 4 Aminu Kano Teaching Hospital, Kano, Nigeria; 5 University of Abuja Teaching Hospital, Abuja, Nigeria; 6 Hasiya Bayero Children’s Hospital, Kano, Nigeria; 7 College of Arts and Sciences, University of Dayton, Dayton, OH 45469 USA; 8 University of Nebraska Medical Center, Omaha, NE 68198 USA

**Keywords:** Infectious diseases, Phylogeny, Evolution, Molecular biology, Diseases, Medical research, Molecular medicine

## Abstract

Infections caused by multi-drug resistant Enterobacterales (MDR-E) are difficult to treat and cause significant mortality, especially in developing countries. This study characterized the phenotypic and genotypic profiles of 49 randomly selected beta-lactam resistant MDR-E previously isolated from patients being managed in hospitals in Nigeria using whole genome sequencing. The study isolates exhibited 85.5% resistance to 3rd generation cephalosporins and 65.3% resistance to carbapenems. The *bla*_TEM-1B_ (29, 59.2%)_,_ *bla*_CTX-M-15_ (38, 77.6%)_,_ and *bla*_NDM-1_ (17, 51.5%) were the most common penicillinase, ESBL, and carbapenem resistant genes across isolates, respectively. Seventeen (45%) of *bla*_CTX-M-15_ was carried on the insertion sequence ISEc9 while *bla*_NDM-1_ (11, 64.7%) were associated with ISEc33. None of the 21 plasmids detected were associated with β-lactamase genes. Higher resistance rates were found in *E. coli* ST-88 (n = 2) and the high-risk ST-692 (n = 2). For *Klebsiella* species, the high-risk clones ST-476 (n = 8) and ST-147 (n = 3) predominated and had higher phenotypic resistance rates and higher number of AMR genes. The mechanisms and pattern of antibiotic resistance differ from patterns previously described with isolates harbouring a wide range of AMRGs. The detection of several chromosomally mediated carbapenemases in our study also represents a significant finding that warrants further investigation to better understand its’ implications for clinical practice and public health. The selected MDR-Es were found to be pan-susceptible to tigecycline and had very low resistance to fosfomycin, suggesting a potential for these as empiric treatments. A surveillance approach incorporating both conventional laboratory techniques and modern molecular techniques is essential for the comprehensive characterization of the emergence and dissemination of antimicrobial resistance in Enterobacterales infections within Nigeria.

## Introduction

Infections with multi-drug resistant Enterobacterales (MDR-E) are a growing global threat. The threat of MDR-E infections, such as extended-spectrum beta lactamase producing Enterobacterales (ESBL-E) and carbapenem-resistant Enterobacterales (CRE), is of great concern in low- and middle-income African countries. These countries have the highest global burden of antimicrobial-resistant (AMR) infections with 114.8 deaths per 100,000 population. Despite this burden, these countries have a striking dearth of medical literature addressing this critical problem in these regions^1^. The resistance profiles of CRE often extends beyond beta-lactam agents to include, aminoglycosides, tetracyclines and chloramphenicol as reported in recent scientific studies^2–4^.

There are several mechanisms of resistance mediating MDR-E, including the production of carbapenemases, AmpC enzymes, and ESBLs^5^. Published data indicate significant differences in the mortality rates of patients with MDR-E infections, such as those caused by CRE versus those resistant by other mechanisms^6–8^. The infections caused by MDR-E are a particular global concern because the encoding genes are often located on mobile genetic elements, such as highly transmissible plasmids and integrons, that may spread among different bacterial species and potentially cause epidemics^9^.

Despite its high burden, there is a paucity of studies characterizing MDR-E strains specifically from Nigeria—the most populous country in Africa^10,11^, which hampers our ability to implement targeted antimicrobial stewardship and epidemiologic control measures. To address this critical gap in knowledge, our study undertakes a comprehensive phenotypic and molecular characterization of beta-lactam resistant MDR-E strains isolated from patients attending six hospitals in Northern Nigeria. By unraveling the mechanisms underlying resistance and understanding the current strain landscape, our research aims to provide essential insights necessary for developing effective interventions, optimizing treatment strategies, and guiding infection control practices in this LMIC setting.

This study characterized contemporary strains of MDR-E to define their phenotypic and genotypic antibiotic resistance profiles. This included elucidating the mechanisms of resistance to cephalosporins and carbapenems, carriage of AMR genes on mobile genetic elements, and correlating sequence types with antimicrobial resistance profiles. We also aimed to determine genetic relatedness of these MDR-E and suggest ways to combat infections caused by these pathogens.

## Results

### Bacterial strains and sample source

We studied 49 non-duplicate Enterobacterales isolates as shown in Table 1. Of these, 83.7% were recovered from blood (n = 41), 12.2% from urine (n = 6), and 4.1% from wound swabs (n = 2). Over half (57.1%, n = 28) of the isolates were *Klebsiella spp*.

**Table 1:** Distribution of Enterobacterales characterized in study.

Genus	Enterobacterales species	n (%)	Isolate source
*Citrobacter spp*	*Citrobacter freundii*	2 (4.1)	BC, U
*Enterobacter spp*	*Enterobacter cloacae subsp. cloacae*	9 (18.4)	BC
	*Enterobacter hormaechei*	1 (2.0)	WS
*Escherichia spp*	*Escherichia coli*	6 (12.2)	BC, U
*Klebsiella spp*	*Klebsiella pneumoniae*	17 (34.7)	BC, U
	*Klebsiella quasipneumoniae*	10 (20.4)	BC
*Serratia spp*	*Serratia ureilytica*	4 (8.16)	BC, U
	Total	49 (100.0)	

Isolate source key: *BC* Blood culture, *U* Urine, *WS* Wound swab.

### Antimicrobial susceptibility testing results

Antimicrobial susceptibility testing was performed for the 49 MDR-E against 17 different antibiotics and also to determine ESBL production. Results are shown in Table 2 and detailed results are presented in Table S1. The prevalence of resistance for third generation cephalosporins among the study isolates was 85.5% to cefotaxime and 80.0% to ceftriaxone. The overall ESBL production among study isolates was 30.3%. Resistance rate to meropenem was 65.3% and carbapenemase production as identified by the mCIM method detected in 55.1% of the isolates.

**Table 2:** Antimicrobial resistance profiles of Enterobacterales by species.

	*Citrobacter spp* (2)	*E. coli (*6)	*Enterobacter spp* (10)	*Klebsiella spp* (28)	*S. ureilytica (3)*	Total resistance n (%)
Amikacin	0 (0.0)	0 (0.0)	7 (50.0)	7 (50.0)	0 (0.0)	14 (28.6)
Gentamicin	2 (100.0)	6 (100.0)	8 (80.0)	26 (92.9)	0 (0.0)	42 (85.7)
Aztreonam	0 (0.0)	6 (100.0)	9 (90.0)	25 (89.3)	0 (0.0)	40 (81.6)
Ampicillin Sulbactam	2 (100.0)	6 (100.0)	10 (100.0)	28 (100.0)	3 (100.0)	48 (100.0)
Piperacillin Tazobactam	2 (100.0)	6 (100.0)	8 (80.0)	23 (82.1)	0 (0.0)	39 (79.6)
Cefotaxime	2 (100.0)	6 (100.0)	9 (90.0)	27 (96.4)	3 (100.0)	47 (95.9)
Cefepime^**#**^	2 (100.0)	6 (100.0)	9 (90.0)	27 (96.4)	0 (0.0)	44 (89.8)
Ceftriaxone	2 (100.0)	6 (100.0)	9 (90.0)	27 (96.4)	1 (33.3)	45 (91.8)
Ceftazidime	2 (100.0)	6 (100.0)	9 (90.0)	27 (96.4)	0 (0.0)	44 (89.8)
Ceftazidime Avibactam	2 (100.0)	4 (66.7)	8 (80.0)	15 (53.6)	0 (0.0)	29 (59.2)
Ceftolozane Tazobactam	N/R	6 (100.0)	9 (90.0)	23 (82.1)	0 (0.0)	38 (80.9)
Meropenem	2 (100.0)	4 (66.7)	8 (80.0)	18 (64.3)	0 (0.0)	32 (65.3)
Colistin*	0 (0.0)	0 (0.0)	9 (90.0)	2 (7.1)	3 (100.0)	14 (28.6)
Tigecycline	0 (0.0)	0 (0.0)	0 (0.0)	0 (0.0)	0 (0.0)	0 (0.0)
Fosfomycin**	0 (0.0)	0 (0.0)	0 (0.0)	7 (25.0)	0 (0.0)	7 (14.3)
Trimethoprim Sulphamethoxazole	2 (100.0)	6 (100.0)	9 (90.0)	27 (96.4)	0 (0.0)	44 (89.8)
ESBL***	0 (0.0)	2 (33.3)	1 (10.0)	12 (44.9)	0 (0.0)	15 (30.6)
mCIM	1 (50.0)	4 (66.7)	8 (80.0)	14 (50.0)	0 (0.0)	27 (55.1)

*Except for colistin, all I breakpoints represented as resistant. ^#^SDD cefepime *E. coli* is reported as R.

**For fosfomycin, NS represented as R.

***ESBL is ≥ 3 times decrease in MIC when cephalosporin is tested alone compared with the addition of an inhibitor.

**Figure 1:**
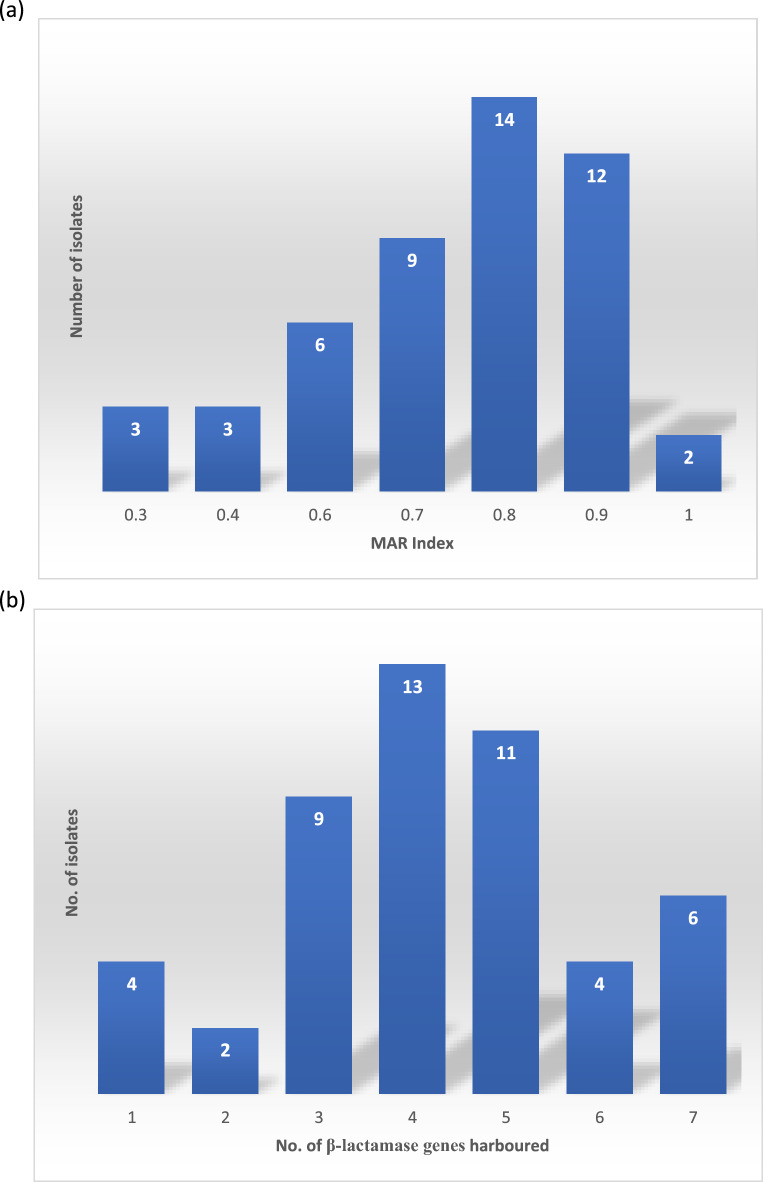
(**a**) Multiple Antibiotic Resistance Index of Enterobacterales in study. (**b**) Isolates with number of β-lactam resistance genes harboured.

In accordance with the selection criteria, all isolates included in the study exhibited resistance to at least one antibiotic within three distinct classes, indicating multidrug resistance (MDR). A total of 14 (34%) of the isolates were resistant to antibiotic agents in at least eight of nine antibiotic classes tested. There was a total of 30 different beta-lactam resistance genes found in this study with the isolates harbouring a minimum of one and a maximum of seven (average of 4) beta-lactamase genes. The multiple antimicrobial resistance (MAR) index and number of β-lactam resistance associated antimicrobial resistance genes (ARGs) harboured by the isolates are shown in Fig. 1 below.

### Antimicrobial resistance genes and the beta-lactamase mobilome

In silico identification of ARGs showed that isolates harboured a wide range of plasmid-mediated and chromosomally encoded resistance genes. Genes encoding resistance to beta-lactam drugs, which include carbapenemases, ESBLs, and *AmpC*s, are shown in the Table 3 below, along with frequency of detection in the Enterobacterales isolates studied.

**Table 3:** Beta-lactam resistance genes detected among studied Enterobacterales.

Antimicrobial resistance gene	Frequency n (%)	Antimicrobial resistance gene	Frequency n (%)	Antimicrobial resistance gene	Frequency n (%)
Penicillinase genes (n = 93)		AmpC and ESBL genes (n = 84)		Carbapenemase genes (n = 33)	
*bla*_TEM-1B_	29 (59.2)	*bla*_CTX-M-15_	38 (77.6)	*bla*_NDM-1_	17 (51.5)
*bla*_SHV-187_	22 (44.9)	*bla*_OXA-1_	27 (55.1)	*bla*_NDM-5_	12 (36.4)
*bla*_OKP-B-15_	9 (18.4)	*bla*_ACT-2_	5 (1.22)	*bla*_OXA-48_	3 (9.1)
*bla*_CMH-3_	8 (16.3)	*bla*_SST-1_	3 (6.12)	*bla*_OXA-181_	1 (3.0)
*bla*_OKP-B-2_	7 (14.3)	*bla*_CMY-2_	2 (4.08)		
*bla*_SHV-67_	3 (6.12)	*bla*_CMY-48_	2 (4.08)		
*bla*_TEM-1A_	3 (6.12)	*bla*_SHV-106_/*bla*_SHV-28_	2 (4.08)		
*bla*_OXA-2_	2 (4.08)	*bla*_CTX-M-14_	1 (2.04)		
*bla*_SHV-11_	2 (4.08)	*bla*_DHA-1_	1 (2.04)		
*bla*_TEM-1D_	2 (4.08)	*bla*_DHA-22_	1 (2.04)		
*bla*_CMH-6_	1 (2.04)	*bla*_SHV-12_	1 (2.04)		
*bla*_MAL-2_	1 (2.04)	*bla*_ACT-5_	1 (2.04)		
*bla*_OKP-A-9_	1 (2.04)				
*bla*_OXA-320_	1 (2.04)				
*bla*_OXA-534_	1 (2.04)				
*bla*_OXA-9_	1 (2.04)				

The most common AMR gene detected was the *bla*_CTX-M-15_ with 38 (77.6%) isolates harbouring the gene. Of these *bla*_CTX-M-15_, 19 (50.0%) of them did not appear on mobile genetic elements (MGEs) and notably, there were no other AMR genes in close proximity to them. In contrast, the other 50% of *bla*_CTX-M-15_ carried on MGEs were rarely carried alone and were associated with several combinations of *bla*_TEM-1A_, *bla*_TEM-1b,_ *aph(3'')-Ib, aac(3)-Iia, sul2*, and *tet(A).* (See Supplementary Table 1) The MGEs carrying the *bla*_CTX-M-15_ were primarily insertion sequences with the most common association being ISEc9 (17), IS5075 (1), and ISKpn43 (1).

The *bla*_OXA-1_ was the next most common ESBL gene being present in 27 (55.1%) isolates. Only two of these were mapped to IS26 insertion sequence while the others 25 (81.5%) were not mapped to MGEs. All *bla*_OXA-1_ genes were located in close association with *bla*_OXA-320,_ *bla*_OXA-534,_ *catB3* and *aac(6')-Ib-cr*.

Thirty-three isolates harboured carbapenemase genes, which were primarily *bla*_NDM-1_ (35.0%) and *bla*_NDM-5_ (25.0%) of which three were susceptible to meropenem (details are shown in the Supplementary Table 1). For the rest of the 46 isolates, the genotypic resistance profiles matched the phenotypic resistance profiles. In addition, the resistance genes identified by Xpert Carba-R testing and WGS were in agreement (the identification of *bla*_OXA-48_ by GeneXpert is inclusive of the *bla*_OXA-48_ variant *bla*_OXA-181_). Most *bla*_NDM-1_ (11, 64.7%) were associated with the insertion sequence ISEc33 and the others not on any MGE. In contrast, *bla*_NDM-5_ were mostly not located on MGEs (10, 83.3%) with the remaining two associated with IS5. The one *bla*_OXA-181_ was associated with the rarely reported insertion sequence ISKpn19 of ISKra4 family.

While no beta-lactamase gene was mapped to plasmids, a total of 21 plasmids in 10 classes were detected in this study for *E. coli* including Col (BS512) (3), ColKP3 (1), IncB/O/K/Z (1), IncFIA (5), IncFIB (AP001918) (5), IncFII (5), IncFII(pAMA1167-NDM-5) (2), IncI1-I(Alpha) (3), IncQ1 (1), IncX3 (3). A total of 18 plasmids were detected among all isolates of *Klebsiella species,* including IncFIB (AP001918) (5), IncFII (5), IncFII(pAMA1167-NDM-5) (2), IncI1-I(Alpha) (3), IncQ1 (1), IncX3 (3).

### Sequence types of Enterobacterales in study

The ST-88 (n = 2) *E. coli* had high MAR index of 0.7 and harboured relatively higher number of AMR genes (average of 21 genes) than other STs. The two ST-692 had lower average MAR index (average = 0.55), and lower total number of AMR genes (n = 17) than ST-88.

Of the 28 *Klebsiella spp* strains, the most common STs were ST-476 (n = 8), followed by ST-147 (n = 3). There were two each of ST-14, ST-1788, ST-25, and ST-429 (Supplementary Table 1). There was one each of ST-101, ST-1224, ST-13, ST-17, ST-336, ST-353, and ST-978. For analysis. the STs were grouped as the commonest ST-476, ST-147, and ‘others’. All ST-147 and ST-476 isolates had at least 17 AMR genes, harboured carbapenemase genes, and were carbapenem resistant unlike the ‘other’ group of STs. The ST-147 group had the highest MAR index average of 7.7, followed by the ST-476 group which had an average MAR index of 7.2 compared to 6.2 average MAR index of the ‘other’ group.

The most frequent *Enterobacter cloacae* ST was ST-1107 (7) with an average MAR index of 6.4, and with one ST-167 that had lower MAR index of 3. There were two strains of *Citrobacter freundii*, (ST-22), both with an average 19 AMR genes.

### Phylogenetic relatedness of Enterobacterales in study

**Figure 2:**
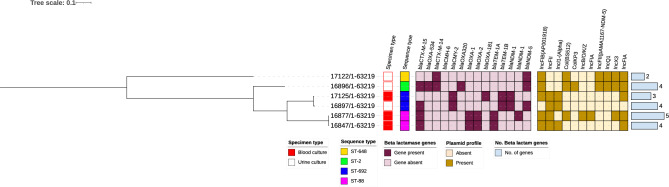
SNP-based phylogenetic relatedness of *E. coli* STs, plasmids, and resistance genes. SNP-based phylogenetic tree of MDR E. coli isolates visualized in Interactive Tree of Life tool (iTOL). SNP-based phylogenetic tree of MDR E. coli isolates visualized in Interactive Tree of Life tool (iTOL). Shown for each isolate from left to right are colour strips for specimen source, and sequence type, heat map for beta-lactamase gens, and bar chart of number of beta-lactamase genes harboured. Tree was midpoint rooted.

The SNP -based phylogenetic relatedness of *E. coli* is captured in the Fig. 2 below indicating the STs, carbapenemase genes, other significant antimicrobial resistance genes, and total number of antimicrobial resistance genes detected. Pairwise SNP difference for all isolates was > 20 implying there were no outbreak strains among the tested strains.

**Figure 3:**
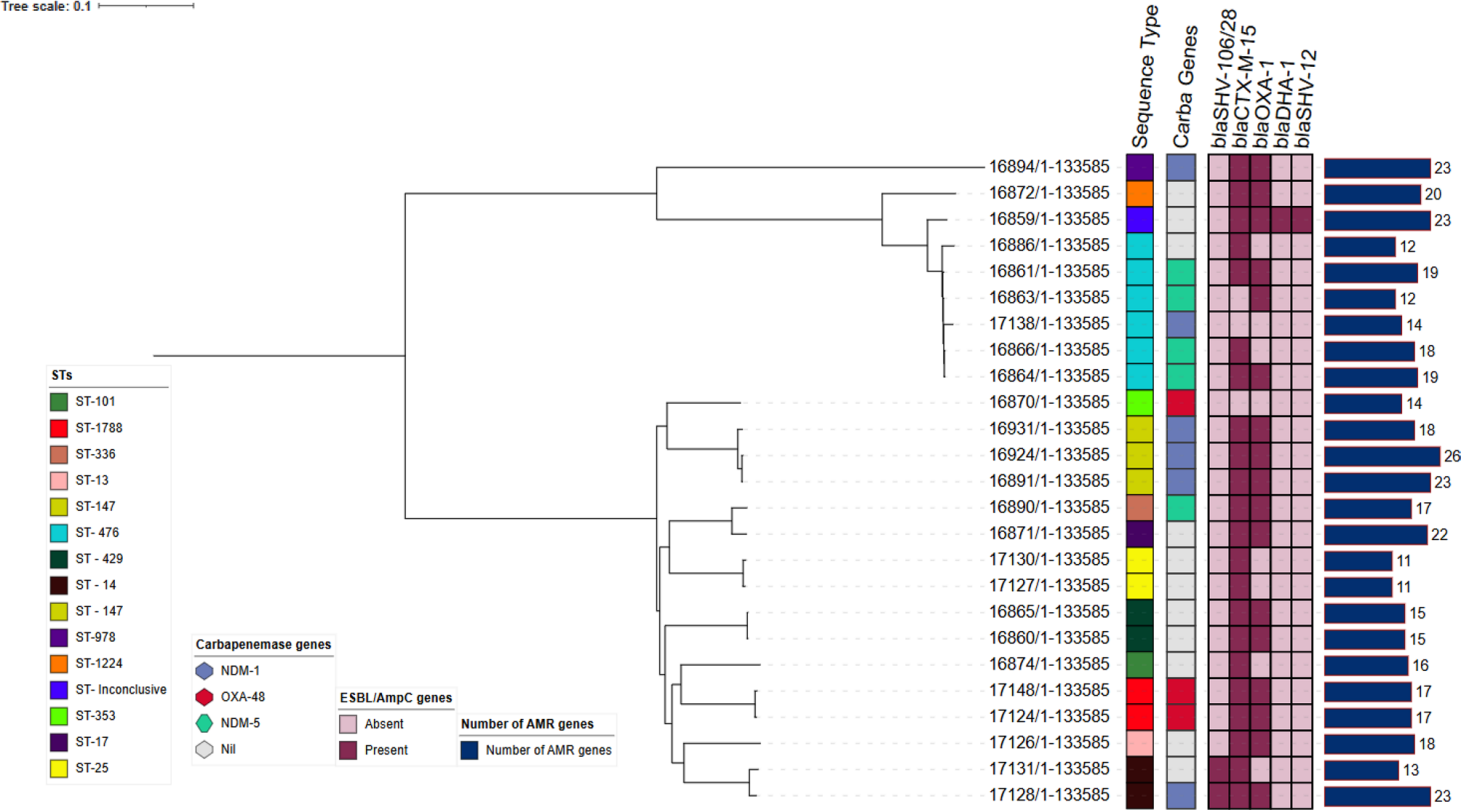
SNP-based phylogenetic relatedness of Klebsiella species STs, plasmids, and resistance genes. SNP-based phylogenetic tree of MDR E. coli isolates visualized in Interactive Tree of Life tool (iTOL). Shown for each isolate from left to right are colour strips for specimen source, and sequence type, heat map for beta-lactamase gens, and bar chart of number of beta-lactamase genes harboured. Tree was midpoint rooted.

None of the *Klebsiella spp* strains in this collection appeared to be outbreak-related as SNP distances were > 20 for all isolates. The phylogenetic tree shown in Fig. 3 depicts the STs, carbapenemase genes, other significant AMR genes, and total number of AMR genes detected. See Supplementary Table 1 for pairwise SNP differences.

**Figure 4:**
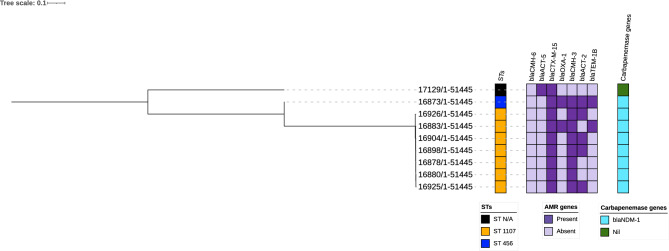
SNP-based phylogenetic relatedness of Enterobacter cloacae STs, plasmids, and resistance genes. SNP-based phylogenetic tree of MDR E. coli isolates visualized in Interactive Tree of Life tool (iTOL). Shown for each isolate from left to right are colour strips for specimen source, and sequence type, heat map for beta-lactamase gens, and bar chart of number of beta-lactamase genes harboured. Tree was midpoint rooted.

None of the *Enterobacter cloacae* strains in this collection appeared to be outbreak-related as SNP distances were > 20 for all isolates. The phylogenetic tree shown in Fig. 4 depicts the STs, carbapenemase genes, other significant AMR genes, and total number of AMR genes detected (See Supplementary Table 1) for pairwise SNP differences.

## Discussion

Antimicrobial resistance, particularly to broad-spectrum carbapenems, is a significant global challenge in medical and public health. Of great concern is the increasing prevalence of antibiotic resistance to beta-lactam antibiotics, which are commonly used as a first-line treatment for infections, particularly in developing nations where they are more cost-effective and easily accessible than other types of antibiotics. This poses a critical threat to infection care and highlights the urgent need for interventions to combat antimicrobial resistance.

Resistance to tigecycline among *E. coli* isolates was not observed in our study, including those strains that were carbapenem resistant. This is contrary to the 23% tigecycline resistance for carbapenem-resistant isolates reported in a surveillance study in the USA^12^. It is worth noting that tigecycline is not typically available or utilized in clinical settings in Nigeria. Except for *K. pneumoniae* which were 75% susceptible to fosfomycin, all other MDR-E studied here were susceptible. While fosfomycin is typically reserved for treatment of uncomplicated UTI, there is an unharnessed potential for use as empiric therapy for infections such as sepsis^13^. Tapping into the potential of tigecycline and fosfomycin especially for settings with high carbapenem resistance is worth investigating further especially because it is cheaper and potentially carbapenem sparing.

A wide array of resistance genes (AMRGs) was found among the Enterobacterales in this study with most of the phenotypic resistance to beta-lactam antibiotics observed, corresponding with expected AMRGs except for the three isolates that harboured carbapenemase genes which were not resistant to carbapenems. This is likely because the genes were not expressed^14^. Genes encoding resistance to penicillins, cephalosporins, and carbapenems were detected both on mobile genetic elements and on the chromosome. The *bla*_TEM-1B_ and *bla*_SHV-187_ were the most common penicillinase genes detected and always occurred with other ESBL or carbapenemase genes. The finding of *bla*_CTX-M-15_ and *bla*_OXA-1_ genes as the more common ESBL genes are in keeping with other studies from the African continent^4,15,16^. The insertion sequence located upstream of the gene and reported as responsible for its mobilization is however different as this study had almost complete ISEc9 unlike previously reported ISEc1^17^. This study findings also contrasts with other studies which have found the *bla*_CTX-M-15_ to be primarily carried on plasmids^15,18^.

In concordance with other studies conducted in similar settings, the *bla*_NDM-1_ and *bla*_NDM-5_ were the most common carbapenemase genes detected^11,19,20^. The strains of Enterobacterales in this study were highly diverse with a significant number of potentially unreported/inconclusive STs that need further analysis. The *E. coli* ST-648, which is considered a high-risk clone mostly reported in hospital settings, and was identified in this setting. Consistent with previous studies, it was recovered from the hospital, was resistant to most antibiotics tested, and harboured a large number of AMR genes^21^. Newly emerging high risk sequence types of *K. pneumoniae* in this study include the ST-101 and ST-147 which had previously been reported as circulating outside the African continent suggests a more global dissemination of this clone^22,23^. The predominant *Enterobacter cloacae*, ST-1107, is a high-risk clone associated with the *bla*_OXA-48_ carbapenemase gene and has been implicated in hospital outbreaks^24^. Similarly, *C. freundii* ST-22 had previously been characterized as being associated with MDR infections in hospital setting^25,26^. While many of the isolates were collected from the same hospitals around the same time period, there was no suggestion of an outbreak strain across all isolates based on ST analyses of individual species although the purposive sampling from a large collection of strains might hinder accurate determination of outbreaks.

While carbapenemases not located on mobile genetic elements are relatively uncommon^27,28^, our study detected these in high numbers outside of MGEs. These chromosomal carbapenemases conferring intrinsic and stable resistance is a concerning development as can lead to more propagation and transmission of these MDR bacteria.

In conclusion, our study has identified high risk Enterobacterales strains that exhibit both phenotypic and genotypic mechanisms of antimicrobial resistance, posing a serious threat to public health in Nigeria. The emergence and dissemination of these multidrug and extensively drug-resistant strains is a major concern, highlighting the urgent need for comprehensive surveillance and monitoring programs which include molecular methods. Our findings emphasize the importance of incorporating both conventional laboratory techniques and modern molecular techniques to provide a comprehensive characterization of antimicrobial resistance in Enterobacterales infections within Nigeria. This approach can facilitate the early detection and prompt response to emerging resistance patterns, allowing for the implementation of effective infection control measures and the development of new therapeutic options.

## Methods

### Study design, sample collection, isolate primary processing and final selection

A total of 49 MDR-E isolates were selected from our archives and processed for this study. These were selected randomly from the archive of over 200 MDR-E isolated from the parent studies of Community-Acquired Bacteremic Syndromes in Young Nigerian Children (CABSYNC) and Community-acquired Pneumonia and Invasive Bacterial Diseases (CAPIBD). These 49 were chosen as a convenience number. All 49 non-duplicate isolates were obtained from clinical specimens collected from the cities of Kano and Abuja. Clinical isolates were collected from patients with suspected blood stream infections, urinary tract infections (UTIs), and wound infections. In Kano, the clinical isolates were obtained from Aminu Kano Teaching Hospital (AKTH), Hasiya Bayero Children’s Hospital (HBCH), and Murtala Mohammed Specialist Hospital (MMSH) while in Abuja, isolates were obtained from the University of Abuja teaching hospital (UATH) and National Hospital Abuja (NHA). Ethical approval was obtained from all the respective hospitals for the primary studies with these isolates collected from 2016 to 2021. Informed consent was collected from all participants and/or their legal guardians. The isolates have been stored in − 80 °C freezers since isolation from original samples.

### Isolation, identification, and antibiotic susceptibility testing of Enterobacterales isolates

Primary processing was dependent on the specimen following standard laboratory procedures with cysteine lactose electrolyte deficient agar-CLED (Oxoid, Basingstoke, UK) for urine, 5% Sheep blood agar (SBA), chocolate agar, and MacConkey agar for positive automated blood culture vials, SBA, and MacConkey agar (Oxoid, Basingstoke, UK) for other specimen types. MacConkey and SBA were incubated in ambient air and Chocolate agar in 5% CO_2_ for 18–24 h at 35–37 °C. The identification and antimicrobial susceptibility testing (AST) of suspected Enterobacterales bacterial colonies were conducted using the Phoenix system (BD Diagnostic Systems, Sparks, MD, US) and interpreted according to Clinical and Laboratory Standards Institute (CLSI) M100 30th Edition guidelines^29^. The control strains *E. coli* ATCC 25922, and *K. pneumoniae* ATCC 700600 were used for quality control. At both IFAIN and Cepheid sites, Xpert^®^ Carba-R (Cepheid, Sunnyvale, CA, USA) was used for identification of five carbapenem resistance genes (*bla*_KPC_, *bla*_VIM_, *bla*_OXA-48_, *bla*_IMP_, and *bla*_NDM_).

### Isolate secondary processing 

A total of 49 MDR-Es identified as above were randomly selected from our archives and shipped to Cepheid (Sunnyvale, CA, USA), for whole genome sequencing. Inclusion criteria was isolates identified as MDR-Es based on predefined resistance patterns and retrieved only from the six hospitals in the primary studies. We excluded duplicate isolates, isolates that were not MDR-Es, isolates with incomplete data, and those collected from hospitals other than the six mentioned here.

Isolates were re-identified using Matrix-Assisted Laser Desorption/Ionization Time-of-Flight Mass Spectrometry MALDI-TOF MS (Bruker Daltonics, Germany) and their AST profiles re-confirmed with MicroScan Detect Neg MIC 2 on the WalkAway 40 SI system (Beckman Coulter, Inc., USA). ESBL confirmation was done according to the MicroScan software rules. Whole genome sequencing (WGS) was conducted and in silico prediction of antimicrobial susceptibility phenotype performed from WGS output. Carbapenem resistance was defined as phenotypic resistance to ertapenem, imipenem, or meropenem (CLSI)^29^. Multi-drug resistance was defined as resistance to at least one agent in three or more antimicrobial classes. The multiple antibiotic resistance (MAR) index for each isolate was calculated using the formula MAR = a/b, where 'a' represents the number of antibiotics the test isolate exhibited resistance to, and 'b' represents the total number of antibiotics to which the test isolates were exposed.

### Whole-genome sequencing for detection and characterization of antimicrobial resistance genes, plasmids, and insertion sequences

Pure cultures of each organism were grown overnight in trypticase soy broth (Hardy Diagnostics, USA) and genomic DNA were extracted using the Qiagen DNeasy Blood and Tissue kit (Qiagen, USA). Sequencing libraries were prepared from extracted DNA using Nextera XT DNA Library Kit (Illumina, USA) and were sequenced using the Miseq instrument with Reagent Kit v3 (Illumina, USA) chemistry. Raw reads were trimmed for quality and de novo assembled using CLC Genomics Workbench v. 21.0.4 (Qiagen Digital Insights, Denmark). Sequence analysis of bacterial isolates included identification of best matching reference using K-mer spectra, multilocus sequence typing (MLST), and drug resistance analysis using CLC Microbial Genomics Module 21.1 (Qiagen) with ResFinder database (downloaded on 2021-05-30, Center for Genomic Epidemiology). All procedures were done in accordance with manufacturer protocols. Prediction of AMR was conducted by using Mobile Element Finder v1.0.3 (2020-10-09) and selecting Acquired Antimicrobial Resistance genes (ResFinder)^30^. We located the mobile genetic elements (MGEs) associated with resistance genes by using Mobile Element Finder with database v1.0.2 (2020-06-09)^30^. Each resistance gene was classified as being carried by a plasmid, other MGE, or as not associated with an MGE. Plasmids were detected using PlasmidFinder-2.0 with threshold for minimum at 95% identity and minimum 60% coverage using draft genome assemblies^31^. The AMR gene substrates were crosschecked on the CARD database website^32^. The high-quality Illumina paired-ends reads generated were assembled de novo into the draft genome sequence for every isolate using the CLC genomic workbench. Quality assessment for genome assemblies was also carried out using the CLC genomic workbench.

### Multi-Locus sequence typing (MLST) of *E. coli*

The PubMLST—Achtman scheme was performed to identify the sequence types (STs) and clonal complexes (CCs) of the isolates^33^. Isolates with 100% match against known MLST alleles were assigned STs and CCs. Those without perfectly matching alleles were identified as unknown or inconclusive STs^34^. In silico MLST-analyses were performed using previously described seven housekeeping genes (*adk, fumC, gyrB, icd, mdh, purA, and recA)*^35^.

### Calling SNPs and inferring phylogeny

The FASTA files generated from WGS were uploaded unto the CSI Phylogeny 4.1 service of Centre for Genomic Epidemiology (https://cge.cbs.dtu.dk/services/CSIPhylogeny/). CSI Phylogeny outputs were generated based on a selected reference sequence for the different Enterobacterales and downloaded as Newick and text files. Thresholds for SNP calling were for depth = 10 ×, for SNP quality − 30, for map quality − 25, and 1.96 for minimum Z score^36^. Visualization annotation, and management of tree files were performed using the interactive Tree of Life tool—iTOL v6 (http://itol.embl.de/itol.cgi). Pairwise SNP differences between genomes were computed to determine if isolates of different origins were related with SNP distances < 21 indicating close relatedness and 21–50 indicating more distant relatedness^37^.

### Ethics statement

The ethics review boards of National Hospital Abuja (NHA) reviewed and gave approval for the study (Approval number NHA/EC/033/2018). The ethics review boards of Hasiya Bayero Children’s hospital, Murtala Mohammed specialist hospital (Approval numbers NHREC/21/08/2008/AKTH/EC/872 and AKTH/MAC/SUB/12A/P-3/VI/972) and Gwagwalada specialist hospital (Approval number FCTA/HHSS/NH/GEN/54/II/128) gave approval for the parent CAPIBD and CABSYNC studies. All bacteria isolates used here were recovered from submitted clinical specimens at the selected hospitals. All procedures we performed were in accordance with the guidelines and regulations of the ethics review board.

### Data collection and analyses

The data collected and recorded in Microsoft Excel was processed and analysed using STATA software (StataCorp. 2019. TX: StataCorp LLC). The distribution of phenotypic and genotypic features was calculated and determined as frequencies and ratios. The relevant data for this study is included within the research article and can also be accessed as supplementary information.

## Supplementary Information

Supplementary Table 1.

## Data Availability

The datasets used and analyzed during the current study are available from the corresponding author upon request. All data generated or analyzed during this study are also included in this published article and its supplementary information files. For whole genome sequencing, raw reads were uploaded to the National Center for Biotechnology (NCBI) database and can be accessed using BioProject ID: PRJNA952997.
